# Soft coral reproductive phenology along a depth gradient: Can “going deeper” provide a viable refuge?

**DOI:** 10.1002/ecy.3760

**Published:** 2022-07-07

**Authors:** Ronen Liberman, Tom Shlesinger, Yossi Loya, Yehuda Benayahu

**Affiliations:** ^1^ School of Zoology, The George S. Wise Faculty of Life Sciences Tel‐Aviv University Tel‐Aviv Israel; ^2^ The Interuniversity Institute for Marine Sciences Eilat Israel; ^3^ Present address: Institute for Global Ecology Florida Institute of Technology Melbourne Florida USA

**Keywords:** coral reproduction, mesophotic coral ecosystem, Octocorallia, Red Sea, *Rhytisma*, seawater temperature, surface brooding

## Abstract

Many species across a wide range of taxa and habitats display phenological shifts and differences in response to both environmental gradients and climate change. Moreover, the wide‐scale decline of numerous ecosystems is leading to increasing efforts to identify zones that might serve as natural refuges from various disturbances, including ocean warming. One such refuge was suggested to be that of the deep coral reefs, but whether depth can provide coral populations with a viable and reproductive refuge remains unclear. Given the global coral‐reef degradation and the key role that corals play as ecosystem engineers, their reproductive ecology has been widely studied. A particular knowledge gap nonetheless exists regarding coral reproductive phenology along a depth gradient. Filling in this gap may uncover the environmental cues that regulate coral reproduction, leading to better predictions of population connectivity, and their possible responses to climate change and other environmental changes. Here, using long‐term in situ observations of the soft coral *Rhytisma fulvum*'s reproductive activity along its entire depth range (0–45 m), we examined the relationship among several environmental factors and the coral's reproductive phenology and activity over five successive annual breeding seasons. Compared with the shallow depths, a lower number of reproducing colonies was found in habitats deeper than 30 m, highlighting possible constraints on coral reproduction at the deeper end of their range. Our results further revealed that an increase in seawater temperature over 1–2‐day intervals during the breeding season correlated with the onset of reproductive activity along the depth gradient, leading to different reproductive periodicities in different depths. These differences suggest that differential temperature regimes and reproductive timing across depth may create intraspecific temporal reproductive segregation, possibly reducing connectivity among populations along a depth gradient. Moreover, we found high variability among years in both the timing of breeding activities and in the level of reproductive synchrony among corals from different depths. Overall, our study questions whether depth can provide a long‐term and viable refuge for corals in the face of global environmental changes.

## INTRODUCTION

Shifts in the timing of seasonal activities of organisms (i.e., phenology) throughout the animal and plant kingdoms are among the most documented ecological responses to environmental changes (Chmura et al., 2018; Poloczanska et al., 2013; Tang et al., 2016). On land, from plants and insects to birds and mammals, a multitude of phenological shifts resulting from climate change have been well documented (Cohen et al., 2018; Tang et al., 2016). Furthermore, comparative studies across gradients of elevation have been useful in elucidating the relationships among climatic conditions and species' ecology and evolution (Chmura et al., 2018; Oldfather et al., 2020). Similar to elevation in terrestrial ecosystems, the environmental conditions influencing marine species phenology, physiology, distribution, and connectivity, vary substantially along depth over relatively short vertical distances (Kahng et al., 2019). Thus, comparative studies across depth may offer an excellent opportunity to elucidate the interactions between environmental or climatic conditions and phenology, and their impact on the ecology and persistence of species. Focusing on a reef‐dwelling soft coral, we took such an approach to study its long‐term reproductive ecology and determine whether depth can provide a viable refuge in times of environmental changes.

The reproductive phenology of marine invertebrates, including seasonal gametogenic cycles, gamete maturation, and timing and synchronicity of breeding activities, is strongly controlled by a suite of environmental cues (Harrison, 2011; Kahng et al., 2011; Mercier & Hamel, 2010). These factors include seawater temperature (Caballes et al., 2021; Howells et al., 2014; Nozawa, 2012), twilight and lunar cues (Lin et al., 2021; Sweeney et al., 2011), wind fields (Sakai et al., 2020; van Woesik, 2010), and solar insolation (van Woesik et al., 2006). Because such cues are defined by physical properties, they exhibit distinct depth‐related gradients. For example, light always decreases exponentially with increasing depth (Kahng et al., 2019); and seasonal thermoclines, upwelling, downwelling, and internal waves may cause significant temperature differences along depth, although those vary both spatially and temporally due to local hydrology, bathymetry, etc. (Kahng et al., 2019; Wyatt et al., 2020). Additionally, wind‐driven waves may affect shallow‐water motion and its temperature (Jokiel & Morrissey, 1993). Accordingly, long‐term reproductive studies, along with monitoring key environmental variables, aimed at capturing both multiannual and between‐habitat variation can provide an in‐depth understanding of marine species' reproductive phenology, and its response to changing climate.

Mesophotic coral ecosystems (MCEs; ~30–150 m depth) have been suggested to function as a natural refuge zone, as organisms inhabiting the MCEs may be somewhat more sheltered from certain stressors (Bongaerts et al., 2010; Lesser et al., 2009), particularly that of high seawater temperatures that lead to coral bleaching (Muir et al., 2017; Pérez‐Rosales et al., 2021; but see Frade et al., 2018; Smith et al., 2016). Consequently, it has been posited that thriving MCEs may aid in the replenishment of degraded shallow reefs by serving as a larval source (Bongaerts et al., 2010). However, determining whether such deep habitats may provide an adequate larval source and viable refuge for corals requires a comprehensive understanding of reproduction along depth. Several studies have already demonstrated spatio‐temporal differences in the periodicity of reproductive activity between shallow and mesophotic coral populations (Feldman et al., 2018; Gori et al., 2007; Holstein et al., 2015; Liberman et al., 2018; Prasetia et al., 2017; Shlesinger et al., 2018). Such phenological differences, however, have been mostly inferred from the absence of gametes in time‐series sampling (e.g., Gori et al., 2007; Prasetia et al., 2017; Shlesinger et al., 2018), with an almost complete lack of direct observations of corals reproducing at depths >20 m (but see notable exceptions by Holstein et al., 2015; Strader et al., 2021; and Vize, 2006). There is therefore a dearth of information regarding the reproductive performance and phenology of corals residing at greater depths, which naturally hinders our ability to understand the overall resilience, functionality, and connectivity across the broader coral‐reef ecosystem.

**FIGURE 1:**
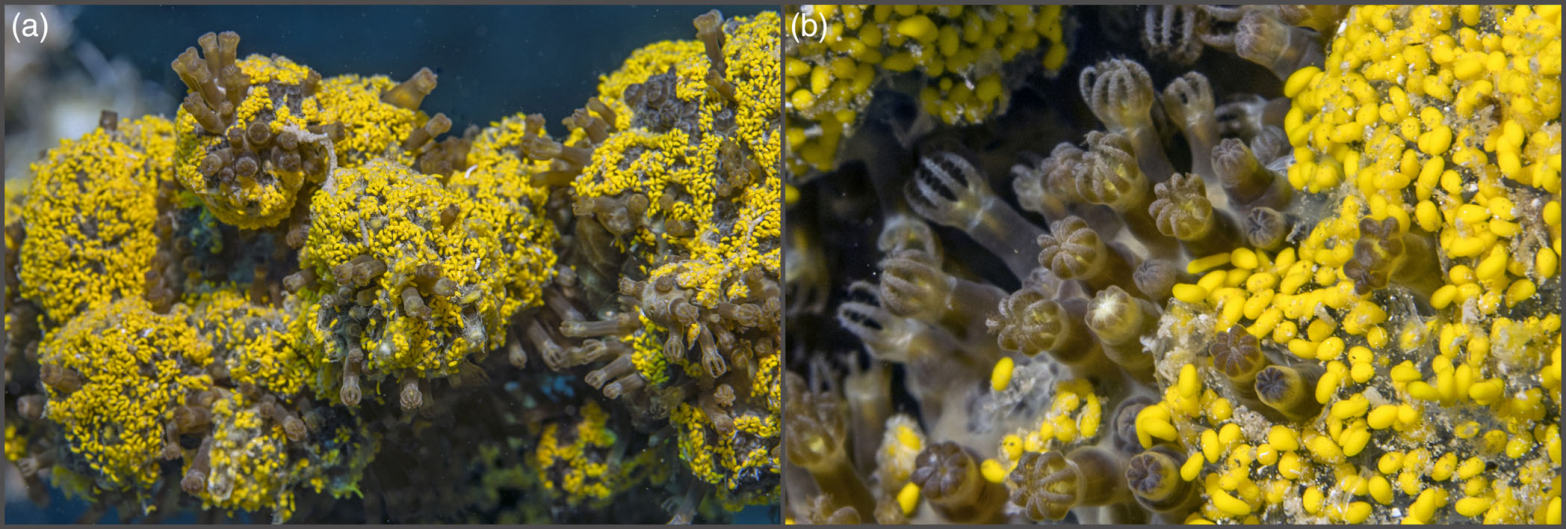
Surface‐brooding *Rhytisma fulvum* colonies on the reefs of Eilat at a depth of ~10 m, featuring lemon‐yellow developing planulae entangled in mucus on the colony surface. (a) View of a colony showing the surface‐brooding event that is easily detected even from a distance. (b) Close‐up view taken on the ~5th day of the surface‐brooding event, showing elongated mature planulae.

Octocorals not only constitute a significant faunistic component in MCEs (Benayahu et al., 2019), but as climate change progresses they might even become more abundant on some reefs than the scleractinian reef builders (Tsounis & Edmunds, 2017). Here, we explored the reproductive phenology and quantified reproductive activity (as the percentage of surface‐brooding colonies) of the zooxanthellate soft coral *Rhytisma fulvum* (Octocorallia, Alcyonacea) along its entire depth of occurrence (0–45 m) and annual reproductive season (June–August) in the reefs of Eilat (Gulf of Aqaba and Eilat, northern Red Sea) over a 5‐year period. This species exhibits external surface brooding in which the fertilized eggs and the subsequent developing embryos are brooded for ~5–7 days on the surface of female colonies, followed by the detachment of planula larvae (Benayahu & Loya, 1983; Kahng et al., 2011; Liberman et al., 2018). In terms of dispersal, like other brooding corals, both the released sperm and the planulae of *Rhytisma fulvum* may disperse to only a few meters away from the parent colony (Warner et al., 2016), due to their negative buoyancy and the planulae's tendency to crawl over the substrate rather than being borne by the currents (Benayahu & Loya, 1983; R. Liberman, personal observation). By contrast, from the temporal aspect of reproductive activity, surface brooding resembles more the brief episodes of broadcast spawners than the continuous breeding periods of most brooders. The striking yellow coloration of *R. fulvum* embryos during surface brooding (Figure 1) facilitates a rapid in situ visual recognition of its reproduction activity, making this species a unique model to study the effects of stress on coral reproduction (Liberman et al., 2021) and to monitor coral reproductive activities, especially in the more challenging depths of the MCEs (Liberman et al., 2018).

Here, we report our 5‐year study of *R. fulvum* reproductive activity across its entire depth of occurrence, together with measurements of relevant environmental parameters. The study aimed to test the hypothesis that reproductive phenology along the depth gradient is correlated to seawater temperature. Using the long‐term data set, we estimated the relative effects of several temperature, lunar phase, and wind metrics on the occurrence of surface‐brooding events along depth. Additionally, we quantified the coral's reproductive activity at different depths to assess possible limitations imposed on populations residing at the extremes of their distribution. We sought to shed further light on the fundamental question ‘Can “going deeper” into the mesophotic depths offer coral populations a viable refuge from disturbances and provide a source of larvae to shallower reef zones?’

## MATERIALS AND METHODS

### Study site and environmental data collection

The study was conducted during 2016–2020 on the reef across from the Interuniversity Institute for Marine Sciences in Eilat (IUI), Gulf of Aqaba and Eilat, northern Red Sea (Israel, 29°30′ N, 34°55′ E). Both the shallow reef (<12 m) and upper MCE (>35 m) feature a flat, mostly hard‐bottomed seabed. In between, there is a steep slope, featuring a patchy hard substrate surrounded by sand and gravel.

Seawater temperature data were obtained using temperature data loggers (HOBO Water Temp Pro v2, Onset Computer Corporation) that have been secured to the reef since 2015 at depths of 5, 15, 30, and 45 m, and programmed to take measurements every 15 min (Appendix S1: Figure S1). The seawater temperature data during June–August of each year (2016–2020) were sorted into several calculated metrics (please refer to the “Data analyses” section below), which were later incorporated into a statistical model. The lunar day at the onset of each surface‐brooding event was also used as an explanatory variable in the model. The daily fluctuations in solar irradiance in the region during the summer months were negligible (Appendix S1: Figure S2) and therefore are not included. To examine the relationship between the prevailing wind and daily fluctuations of the seawater temperature, wind speed data were obtained from a meteorological station, located 10 m above sea level, at the IUI. Data courtesy Israel National Monitoring program in the Gulf of Eilat (https://iui-eilat.huji.ac.il/Research/NMPMeteoData.aspx). Pearson correlation tests were used to examine the relationship between the daily mean wind speed (m/s) and daily change in seawater temperature in the different depth zones.

### Reproduction surveys along depth

In situ monitoring of the onset and duration of surface‐brooding events commenced each year in early June, prior to the predicted reproductive period of *R. fulvum* (Benayahu & Loya, 1983), and continued until early August, which marks the end of the reproduction season for this species (Liberman et al., 2018). Surveys were performed at 2–4‐day intervals, starting at 45 m depth and ascending to the shallowest depth. Following each in situ observation of initiation of surface brooding at any given depth, daily surveys were then conducted for 8–10 successive days, while recording the depth range of each surface‐brooding event. In each event, numbers observed were between dozens and a few hundred surface‐brooding colonies. During June–July 2016, only the onset and duration of surface‐brooding in the shallow reef and upper MCE were recorded. In addition, during the following 4 years (2017–2020), the activity of surface‐brooding colonies was also quantified at five designated depth intervals: 0–10, 10–20, 20–30, 30–40, and 40–45 m. The latter two depths are regarded as upper MCEs; whereas the shallowest depth colonies were at 0.5 m, just below the sea surface. During each surface‐brooding event, three or four belt transects (30 m long and 1 m wide) were randomly deployed at each depth interval and the number of colonies either with or without surface‐brooded planulae was recorded. The surface‐brooding activity was calculated by dividing the number of surface‐brooding colonies by the total number of colonies within each depth interval.

### Data analyses

To determine which environmental variables best explained the temporal differences in the timing of surface‐brooding onset along depth, a generalized linear model (GLM) with a binomial link function (i.e., logistic regression), was fitted. For each of the surface‐brooding events from all 5 years of the study, *R. fulvum* colonies found at each depth interval were scored for the presence or absence of surface‐brooded embryos (1 or 0: brooding occurred or not, respectively). In situ seawater temperature (°C) measurements taken every 15 min at the different depths were used to calculate the daily mean, daily maximum, daily range (maximum–minimum), daily change (current day minus previous day daily mean), 2‐day change (current day mean minus daily mean of previous 2 days), 3‐day change (current day mean minus daily mean of previous 3 days), and 5‐day running average rate (i.e., the regression slope of the change in daily mean during 5 consecutive days). Lunar day at the onset of each of the surface‐brooding events was recorded (with lunar day 0 indicating a new moon) and used as an indication of lunar periodicity in the model. The lunar day variable was assumed to follow a linear circular regression (periodic regression), and as such it was expressed in sine and cosine terms, following its conversion into radians as the product of lunar days and 2π (DeBruyn & Meeuwig, 2001). The cosine term describes phase shifts near 0° and 180° (i.e., new and full moons) and the sine describes phase shifts near 90° and 270° (i.e., first and third quarters). Continuous variables were tested for collinearity using a Pearson correlation test. Here, 1, 2, and 3‐day temperature changes were found to be highly correlated, as were daily maximum and daily mean and range (*r* > 0.65 for all; Appendix S1: Figure S3). Consequently, we performed model selection using Akaike's Information Criterion (AIC) to determine which variables best fitted the model. According to the AIC, 2‐day change, daily mean, daily range, and 5‐day running average rate were chosen for the final model, whereas 1‐day and 3‐day changes and daily maximum were omitted. Following the exclusion of correlated variables, a full model including all remaining variables (without interactions) was constructed and a backward model selection procedure was performed. The best‐fitting model was selected based on AIC scores. As a final stage, we introduced interaction terms in the best‐fitting model, which was then validated by visual inspection of residual plots, QQ plots, and residuals/leverage plots.

Differences in surface‐brooding activity among *R. fulvum* colonies at the five depth intervals from 0 to 45 m were assessed using a generalized linear mixed model (GLMM) with a binomial link function. To account for the multiple surface‐brooding events within the same year, we included year and sequential order of events within that year (i.e., first, second, or third event) as random effects, and depth as the fixed effect. Model validation was performed by visual inspections as described in the previous paragraph. In 2018, no surface‐brooding colonies were found deeper than 30 m and therefore no quantifications were performed below this depth. To accommodate these missing data in the model, the number of non‐brooding colonies was estimated based on the average number of colonies monitored in the transects at these depth intervals during the other 4 years. The significance of differences in surface‐brooding activity across depth was estimated using the Wald statistic, which approximates the chi‐squared (χ^2^) distribution. Pairwise comparisons with Bonferroni correction were performed to compare the surface‐brooding activity among the five depth intervals. All data analyses and graphics were produced using R v.4.05 (R Core Team, 2019) complemented with the packages *lme4*, *MuMin*, *emmeans*, and *ggplot2*.

## RESULTS

### Depth‐related spatial and temporal reproductive dynamics

**FIGURE 2:**
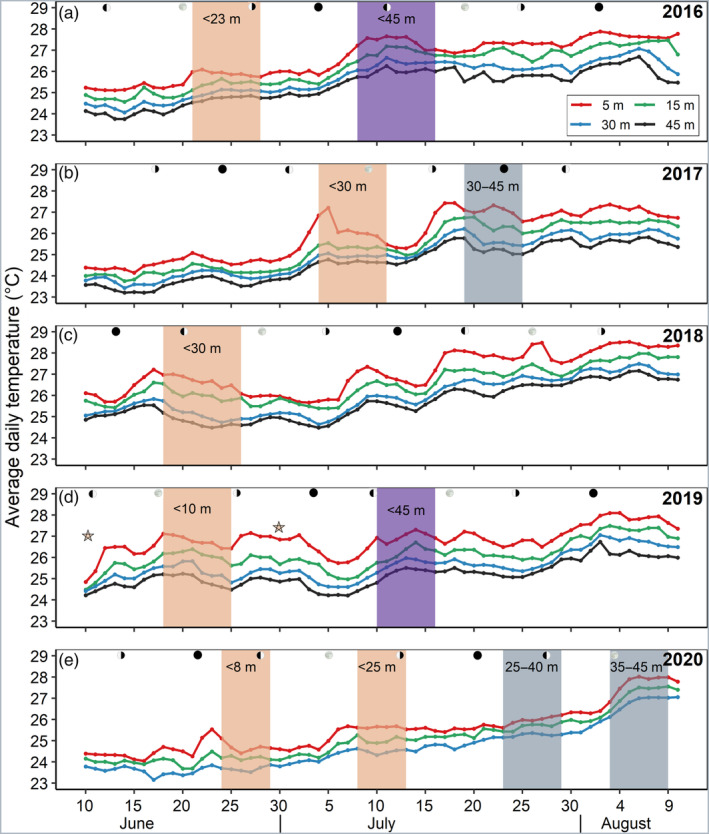
Surface‐brooding periodicities and mean daily seawater temperatures from shallow to mesophotic depths during June–August 2016–2020. Panels (a–e) represent different years, with colored lines indicating mean daily seawater temperatures at four different depth intervals according to the legend in (a). Shaded background represents the timing of surface‐brooding events with those occurring exclusively at shallow depth or mesophotic coral ecosystem depicted in orange or gray, respectively, and in purple when occurring throughout the depth gradient. Lunar phases indicated by black circles (new moon) and white circles (full moon). Asterisks in (d) represent minor surface‐brooding events that occurred at shallow depth (<10 m).

The reproductive phenology of *R. fulvum* in terms of timing of onset, synchronicity, and activity varied between years and depths during this 5‐year study (Table 1 and Figure 2). Whereas the annual onset of the surface‐brooding events consistently started each year in the shallow reef and then proceeded to the deeper waters, there was a large variation in both periodicity and synchrony of the events among the years. In both 2016 and 2019, two surface‐brooding events were observed, in which the first event was limited to colonies at depths shallower than 23 and 10 m, respectively, and the second event was simultaneous along the entire depth range (Figure 2a,d). By contrast, in both 2017 and 2020, the surface‐brooding events were separated in both time and depth, resulting in an apparent reproductive segregation among the colonies residing at different depths (Figure 2b,e); whereas in 2018 no surface‐brooding took place in the upper MCE (Figure 2c).

**TABLE 1:** Pairwise comparisons of *Rhytisma fulvum* surface‐brooding activity at five depth intervals

Depth intervals (m)	Surface‐brooding probability	SE	Asymptotic minimal CI	Asymptotic maximal CI
0–10^a^	0.255	0.038	0.17	0.366
0–20^a^	0.273	0.047	0.17	0.408
20–30^a^	0.24	0.046	0.141	0.377
30–40^b^	0.086	0.027	0.038	0.184
40–45^b^	0.042	0.019	0.013	0.129

*Notes*: Surface‐brooding probability and standard error (SE) represent the estimated marginal means of the tests. ^a,b^Different lettering for depth intervals indicates significant differences of surface‐brooding probability tested with a generalized linear mixed model (GLMM) with a binomial link function. CI, confidence interval.

The first major surface‐brooding event in 2016 took place from 21 to 27 June, initiated 1 day after the full moon and was limited to the upper 23 m (Figure 2a). The following event in that year occurred 10 days later, from 8 to 15 July, initiated 3 days before the lunar first quarter and recorded along the entire depth gradient (Figure 2a). Similarly, in 2019, the first major event occurred from 18 to 25 June, initiated 1 day after the full moon and limited to the upper 12 m; whereas the second event, which took place 15 days later, from 9 to 15 July, was initiated on the day of the lunar first quarter and took place along the entire depth range (Figure 2d). In 2017, the first surface‐brooding event occurred from 4 to 11 July, initiated 4 days after the first lunar quarter and took place only at the shallow depths of 0–30 m (Figure 2b). It was then followed by a second event, 15 days later, from 19 to 26 July, initiated 3 days after the third lunar quarter and took place only in the upper MCE (Figure 2b). In 2018, the single surface‐brooding event occurred only down to a maximal depth of 30 m, from 18 to 25 June, initiated 2 days before the first lunar quarter (Figure 2c). Interestingly, in 2019, in addition to the two major events noted above, two additional minor surface‐brooding events were observed, confined to only a few uppermost shallow‐water colonies (indicated by the asterisks in Figure 2d). In 2020, however, multiple surface‐brooding events were recorded at distinct depth intervals, with the first two taking place at a depth range of 0–8 m and 0–25 m (Figure 2e; 24–29 June and 7–12 July, respectively). Later that year, two additional events took place but only in the upper MCE, at depths of 25–40 m and 35–45 m (Figure 2e; 23–29 July and 3–8 August, respectively).

**FIGURE 3:**
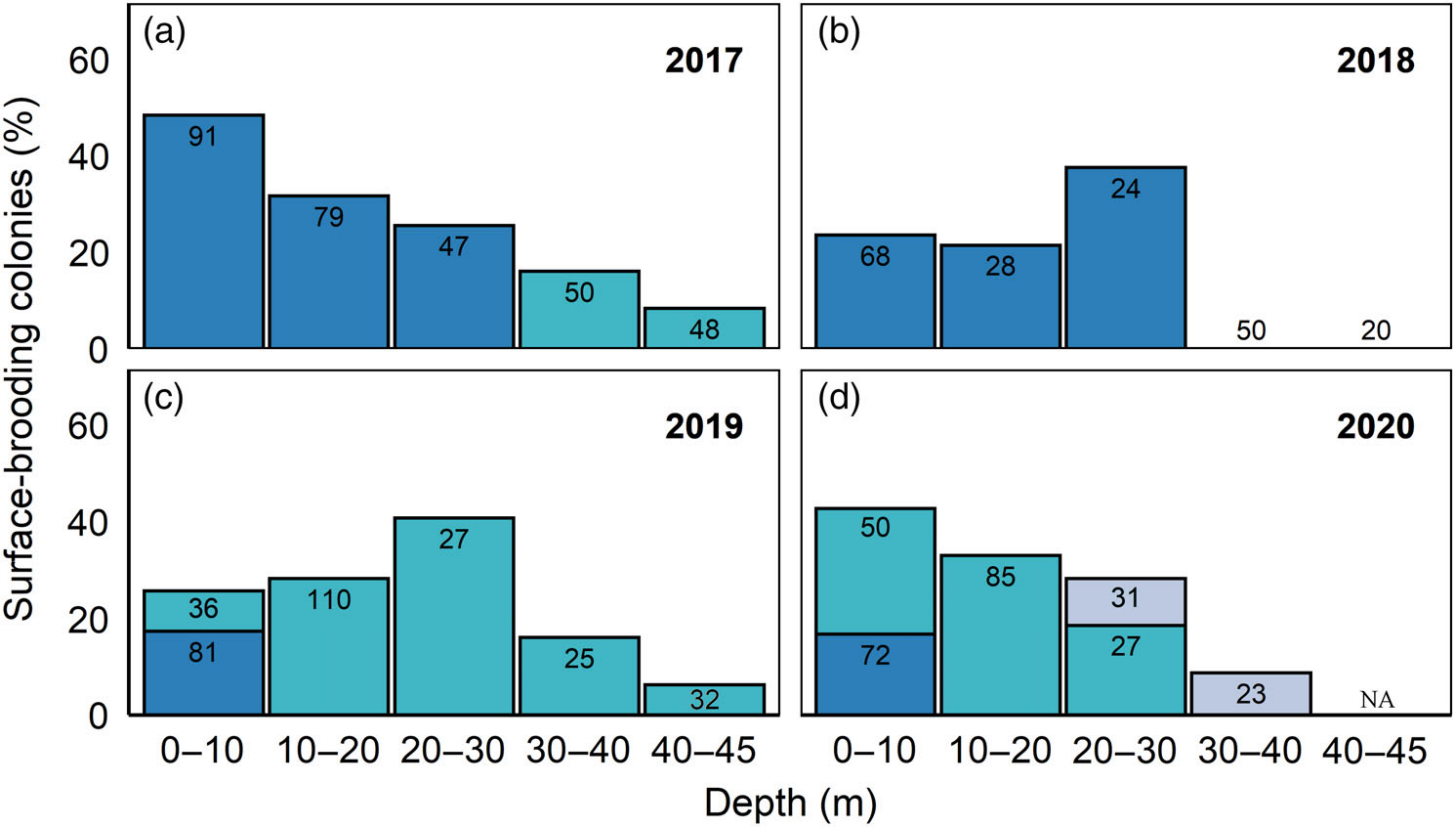
Bar plots represent surface‐brooding activity of *Rhytisma fulvum* observed on the reef. Different panels (a–d) represent different years and colored bars representing sequential order of annual events, with the first events in blue, second in turquoise, and third in gray. Numbers at the top of the bars are the total number of colonies observed at each event and depth interval.

Surface‐brooding activities during 2017–2020 were significantly affected by depth (GLMM: Wald statistic = 31.9, *df* = 4, *p* < 0.001; Table 1 and Figure 3). Pairwise comparisons among the different depth groups revealed that the surface‐brooding activity at the three shallower depth intervals was significantly higher than at the two other, deeper ones (Table 1), with no significant differences among the three shallow‐depth intervals (0–10, 10–20 and 20–30 m), or among the upper MCE ones (30–40 and 40–45 m; Table 1).

### Modeling reproduction and environmental parameters

**FIGURE 4:**
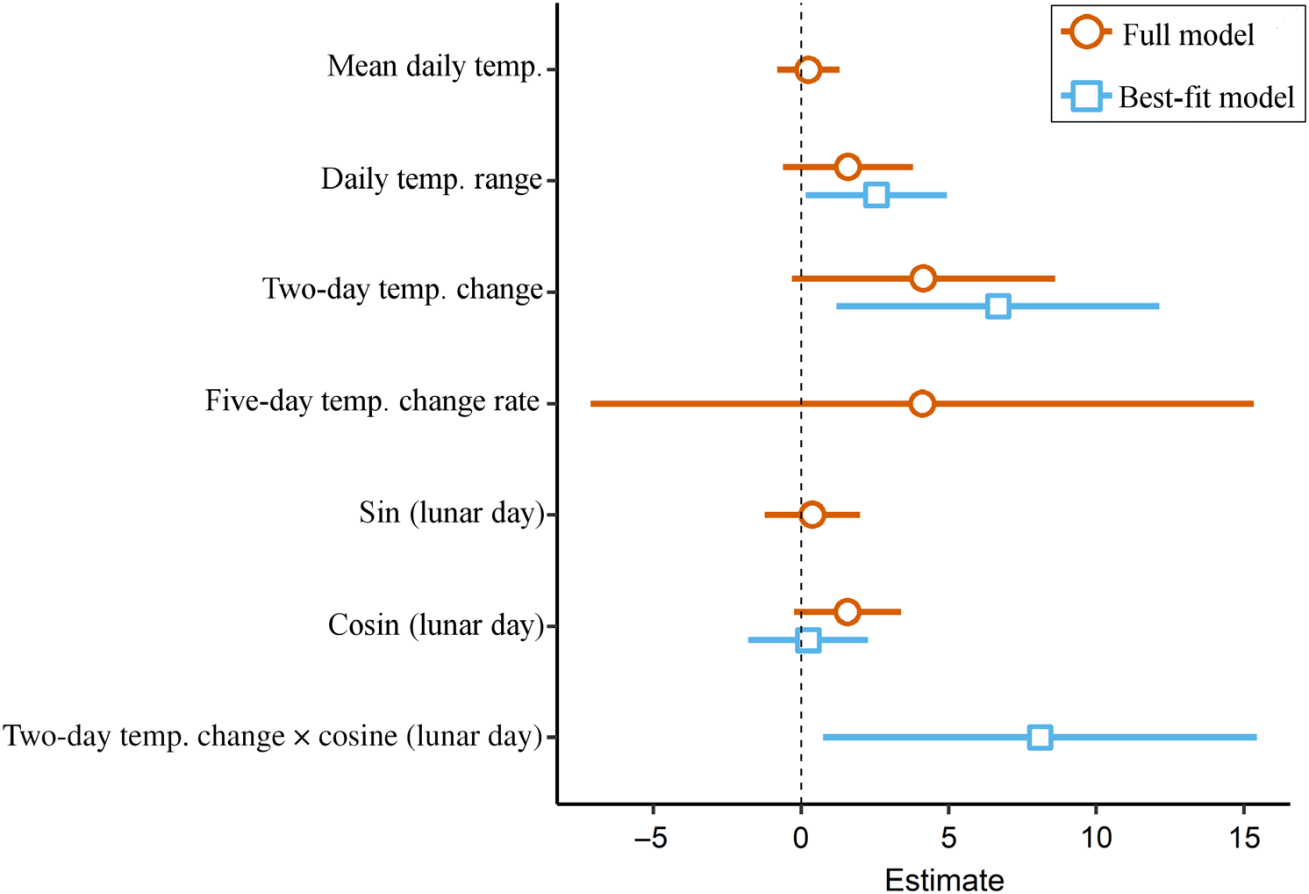
Estimated effect sizes of the generalized linear model (GLM) coefficients, showing contribution of modeled environmental variables in explaining the timing of onset of *Rhytisma fulvum* surface‐brooding events. Coefficients of the initial full‐parameter GLM are shown in orange and coefficients of the best‐fit GLM in blue.

**FIGURE 5:**
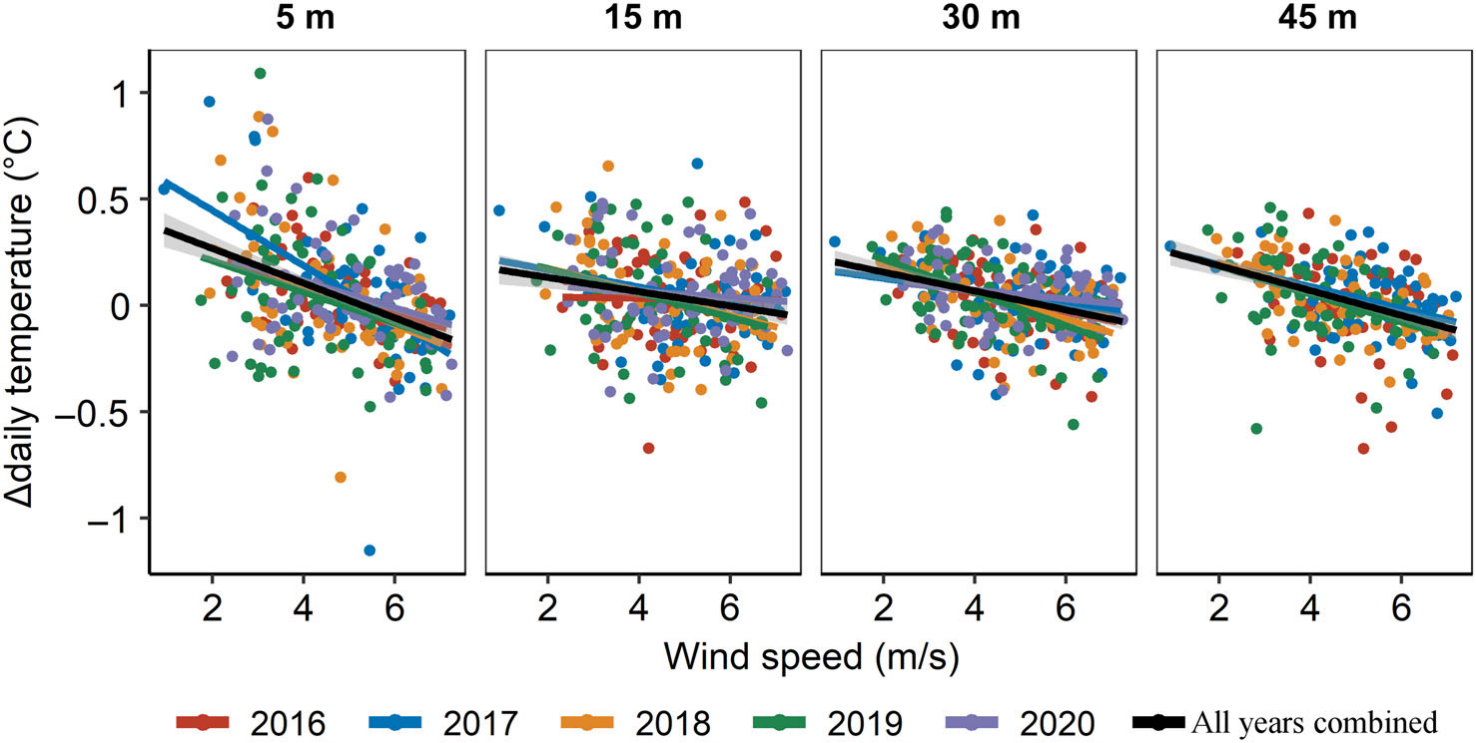
Relationships between wind speed and daily change in seawater mean temperature at four different depth zones during June–July 2016–2020. Colored dots and trend lines represent different years, with black lines representing the multiannual average linear trend.

Maximum daily temperature demonstrated a high collinearity with the mean daily temperature and daily range (Pearson correlation test, *r* = 0.96 and 0.7, respectively; Appendix S1: Figure S3) and thus was excluded from further analyses. Due to the nestedness of the 1, 2, and 3‐day temperature difference variables, they also showed collinearity (Appendix S1: Figure S3) and thus warranted a selection procedure. Each variable was tested in a full model together with all other variables. The model including the 2‐day temperature difference presented the lowest AIC score and this variable was therefore selected. Based on the backward model selection, the final best‐fit GLM included the daily temperature range, 2‐day temperature difference, cosine of lunar day, and the interaction between the latter two factors (Figure 4). Notably, the addition of an interaction term between the 2‐day temperature difference and cosine of lunar day considerably improved the model (compared with the same one without interactions), and accounted for a larger proportion of the explained variation (*R* ^2^_Nagelkerke_ = 56% with interaction compared with 44% without, ΔAIC between the two models = 3.8; Appendix S1: Table S1). The interaction between the 2‐day temperature difference and cosine of lunar day contributed significantly to the best‐fit model (β = 8.09, *p* = 0.0031). However, each of these parameters individually contributed differently to the model. The 2‐day temperature difference factor had a significant effect on the timing of onset of surface‐brooding (β = 6.67, *p* = 0.0017), whereas the lunar day factor did not (β = 0.24, *p* = 0.82). Daily temperature range was also positively related to the onset of surface‐brooding (β = 2.55, *p* = 0.036). Last, the correlation between daily changes in wind speed and seawater temperature along depth during the annual breeding season (June–August) revealed an unexpected pattern: whereas the temperatures at 5 and 45 m depths consistently showed an inverse correlation with wind speed throughout the entire duration of the study, at the 15 and 30 m depths the correlations were less consistent among the years (Figure 5; Appendix S1: Table S2).

## DISCUSSION

In the face of the ongoing biodiversity and climate crises (Hughes et al., 2017; Kleypas et al., 2021; Pereira et al., 2010), scientists are increasingly searching for zones that might serve as natural refugia: areas that might safeguard species or entire ecosystems against certain disturbances, including global warming (Fine et al., 2013; Keppel et al., 2012; McLaughlin et al. 2017; Morelli et al., 2020; van Woesik et al., 2012). Mesophotic coral ecosystems have been suggested to act as such refuge zones, buffered from a variety of disturbances, and as a source of planulae that may replenish degraded shallow reefs (Bongaerts et al., 2010; Lesser et al., 2009). Indeed, mesophotic depths might offer some level of protection, for example, from the coral bleaching that results from anomalously high seawater temperatures (Muir et al., 2017; Pérez‐Rosales et al., 2021). However, the extent to which MCEs represent marginal populations that may be subjected to limiting environmental conditions that affect the fitness and viability of populations, remains unclear.

Our 5‐year study of the reproductive activity of the soft coral *Rhytisma fulvum*, along its entire depth range, has revealed that whereas the proportion of reproductive colonies remained similar across the upper 30 m of the reef, it considerably decreased toward the deeper edge of the species' range in the mesophotic‐depth zones (Table 1 and Figure 3). Additionally, it appears that, in some years, mesophotic‐depth populations may fail to reproduce entirely. These findings substantiate the accumulating evidence of reduced coral reproductive capabilities in MCEs compared with their shallow‐water conspecifics (Bloomberg & Holstein, 2021; Feldman et al., 2018; Gori et al., 2007; Liberman et al., 2018; Prasetia et al., 2017; Shlesinger et al., 2018; Tsounis et al., 2006). The current findings further revealed the plasticity in the onset of coral breeding events, which were found to be related to rapid increases in ambient seawater temperatures. Accordingly, due to differences in the temperature regime along the depth gradient, corals residing at different depths were observed to commence their reproductive activity on different days or weeks, leading to temporal reproductive segregation and inconsistent reproductive synchronicity between years and depths. Together, these findings demonstrate the constraints imposed by depth‐related environmental conditions on coral reproduction. Diminished reproductive activity and phenological differences further challenge the feasibility of mesophotic reefs, providing a long‐term refuge zone, as these greater depths seem to constitute a population sink more than a source of propagules for shallower reefs. Moreover, the regulation of critical milestones in annual coral reproductive cycles by seawater temperature raises concerns that, as the planet's climate continues to change and the oceans continue to warm, the timing of coral breeding activities might become disrupted or potentially shifted to outside optimal breeding periodicities.

Several environmental factors, such as seawater temperature (which is also affected by wind and solar insolation), may demonstrate strong gradients along depth (Kahng et al., 2019), and may therefore lead to the temporal disparities observed in the timing of coral reproduction along depth (Gori et al., 2007; Liberman et al., 2018; Prasetia et al., 2017; Shlesinger et al., 2018). The seasonal dynamics of seawater temperature constitute a major environmental factor that is affecting coral reproductive phenology, including the acceleration of gamete maturation and regulation of breeding periods (Harrison, 2011; Howells et al., 2014; Keith et al. 2016). Accordingly, several earlier findings of differences in the timing of coral reproduction between shallow‐ and mesophotic‐depth corals have been suggested to be influenced by differences in temperature regimes (Shlesinger & Loya, 2019a and references therein). The current findings provide further evidence that differences in temperature along depth may indeed explain the marked differences in reproductive periodicity between shallow and mesophotic populations (Figure 2). As wind fields can also be related to coral reproduction seasonality (van Woesik, 2010), we examined the relationship between seawater temperature and wind speed, and found that daily changes in seawater temperature consistently exhibited a significant inverse relationship with wind at 5 m and 45 m; but, surprisingly, not at 15 and 30 m (Figure 5; Appendix S1: Table S2). Strong winds generate mixing of the surface seawater and therefore influence seawater temperatures (Shang et al., 2008) and prevent its warming during the daytime; whereas during the cooler nights such winds may lower the surface water temperature, resulting in deepening of the thermocline (Manasrah et al., 2019). By contrast, light winds may increase stratification of the water column, resulting in the accumulation of heat flux and a rapid increase in seawater temperature, mostly of the surface layer. Additionally, light is the most prominent source of energy for autotrophic corals, absorbed via their symbiotic, photosynthetic algae (Falkowski et al., 2006); and, accordingly, solar insolation also plays a critical role in coral reproductive cycles (van Woesik et al., 2006). The results of our study demonstrate that surface‐brooding in the MCE population consistently lags behind that of the shallow one (Figures 2 and 4), which might also be due to the exponential reduction in light intensity with depth (Kahng et al., 2019). This lag in timing could result from the longer time needed to acquire the photosynthetically‐derived assimilates necessary for gonad development and maturation by corals in deeper habitats, compared with those inhabiting the well illuminated shallow depths (Ferrier‐Pagès et al., 2021).

The temporal reproductive segregation along depth found during most years of the present study indicates a consequent diminished potential for cross‐fertilization and connectivity among populations from different depths. Parentage analysis aimed at estimating sperm dispersal distance in a brooding stony coral has shown that most mating occurred among corals residing within a 0–10 m distance from each other (Warner et al., 2016). Accordingly, we hypothesized that most of the reproductive events we observed were the result of fertilization among nearby colonies at the same depth, leading to depth‐dependent assortative mating. Moreover, although brooding species might be capable of migrating across depth during the larval stage, the planulae usually settle in the vicinity of the parent colony (Warner et al., 2016), leading to intraspecific genetic divergence even across relatively short distances (Bongaerts et al., 2017; Prada & Hellberg, 2021; but see Bongaerts et al., 2017; Prada & Hellberg, 2021; Serrano et al., 2016; Thomas et al., 2020). It would seem that coral planulae may be less successful in migrating from deeper habitats to shallow ones and establishing there, than the other way around (Prada & Hellberg, 2021; Rippe et al., 2021; Serrano et al., 2014; Shlesinger & Loya, 2021). Indeed, the current findings are consistent with several studies suggesting that population connectivity along depth may be relevant only for some depth‐generalist species but not for all, or not in all biogeographic regions (Bongaerts et al., 2017; Drury et al., 2020; Liberman et al., 2018; Rippe et al., 2021; Scucchia et al., 2021; Serrano et al., 2014; Shlesinger et al., 2018; Shlesinger & Loya, 2021). Taken together with our findings of reduced reproductive activity at mesophotic depth, it seems less likely that migrating into the deeper MCEs can provide corals with a long‐term viable refuge that will enable them to successfully replenish and sustain shallow‐water populations.

The impact of global climate change on marine organisms is projected to increase (Poloczanska et al., 2013). As many coral reefs worldwide continue to decline, it has been suggested that mesophotic coral reefs may aid in the recovery of degraded shallow reefs (Bongaerts et al., 2010; Lesser et al., 2009). Nonetheless, the nature of the environmental factors that influence coral reproductive capacity along depth is underexplored. Consequently, our ability to evaluate the potential contribution of mesophotic populations remains limited. Although in some years we found that reproductive events that were synchronized along the entire depth range of the studied species, in other years the MCE population may not have reproduced at all or reproduced during a different period and to a lesser extent than that of the shallow population. Both the reduced number of reproductive colonies and a lower reproductive synchronicity are negatively affecting fertilization success, larval production, and subsequent recruitment (Hughes et al., 2019; Knowlton, 2001; Shlesinger & Loya, 2019b). Together with *R. fulvum*'s decreasing abundance at mesophotic depths (Shlesinger & Loya, 2021), the current findings further point to a higher dependency of the MCE population on larval subsidy from shallow‐depth populations, rather than vice versa. Our work demonstrates how long‐term observations of coral reproduction, alongside measurements of environmental parameters, can provide valuable insights into the ultimate cues that regulate coral reproductive phenology. Not only can such studies benefit conservation and management efforts, but they can also contribute to a better understanding of marine species' phenological responses and their consequences in an era of rapid local and global changes.

## AUTHOR CONTRIBUTIONS

Ronen Liberman and Tom Shlesinger co‐led the study, collected and analyzed the data and co‐wrote the first draft. Yehuda Benayahu and Yossi Loya edited the manuscript and provided resources. Yehuda Benayahu supervised the study. All authors contributed to the revisions.

## FUNDING INFORMATION

This research was supported by the TASCMAR project (Tools and Strategies to access original bioactive compounds from cultivated marine invertebrates and associated symbionts), which received funding from the European Union's Horizon 2020 research and innovation program under grant agreement No. 634674; and by the Israel Cohen Chair in Environmental Zoology to Y. Benayahu. The research complied with a permit issued by the Israel Nature and Parks Authority. This study was also supported by fellowships from the Israel Taxonomy Initiative, Rieger Foundation, and IUI to T. Shlesinger.

## CONFLICT OF INTEREST

The authors declare no conflict of interest.

## Supporting information


Appendix S1

## Data Availability

Supporting data and script (ronenliberman, 2022) are available in Zenodo: https://doi.org/10.5281/zenodo.6391022.
