# Supplementary material for: Soft coral reproductive phenology along a depth gradient: Can “going deeper” provide a viable refuge?
Source: Ecology. 2022 Jul 7;103(9):e3760. doi: 10.1002/ecy.3760 (PMC9540190; doi:10.1002/ecy.3760)
Supplement: Supplementary file 1 — Appendix S1 [file ECY-103-e3760-s001.pdf]

Appendix S1.

Journal: *Ecology*

Soft coral reproductive phenology along a depth gradient: Can "going deeper" provide a viable refuge?

Ronen Liberman[1,2*], Tom Shlesinger[1,3], Yossi Loya[1], Yehuda Benayahu[1]

[1]School of Zoology, The George S. Wise Faculty of Life Sciences, Tel-Aviv University, Tel-Aviv 69978, Israel.

[2]The Interuniversity Institute for Marine Sciences, Eilat, 8810302, Israel.

[3]Current address: Institute for Global Ecology, Florida Institute of Technology, Melbourne, FL 32901, USA

Table S1. Pearson correlation test coefficient (r) and significance (α = 0.95) of wind speeds (m/sec) and daily temperature change (°C) at four different depths during June–August, 2016–2020.

Year / Depth	2016		2017		2018		2019		2020	
	r	P. value	r	P. value	r	P. value	r	P. value	r	P. value
**5 m**	-0.43	0.003	-0.58	<0.001	-0.44	<0.001	-0.33	0.018	-0.37	0.024
**15 m**	-0.02	0.89	-0.28	0.052	-0.35	0.006	-0.32	0.018	-0.1	0.9
**30 m**	-0.3	0.069	-0.24	0.058	-0.51	<0.001	-0.52	<0.001	-0.19	0.811
**45 m**	-0.36	0.023	-0.43	0.001	-0.56	<0.001	-0.43	0.002	NA	NA

Table S2. Estimated coefficients of the generalized linear models (GLM), showing contribution of environmental variables in explaining the timing of onset of *Rhytisma fulvum* surface-brooding events. Significant variables (α =0.95) are marked in bold.

Predictors	Best-fit model			Full model		
	Odds Ratios	*CI*	*p*	*Odds Ratios*	*CI*	*p*
(Intercept)	0.06	0.00 – 0.61	**0.033**	0.00	0.00 – 18330682.01	0.484
Daily temp. range	12.78	1.49 – 203.37	**0.036**	4.93	0.63 – 59.83	0.156
Two-day temp. change	788.51	9.44 – 725947.05	**0.017**	62.97	1.01 – 9713.00	0.069
Cos (lunar day)	1.27	0.15 – 9.93	0.817	4.83	0.88 – 37.70	0.088
Two-day temp. change * cos (lunar day)	3266.21	5.21 – 21776176.20	**0.031**			
Mean daily temp.				1.28	0.44 – 3.91	0.650
Five-day temp. change rate				60.49	0.00 – 9295362.50	0.474
Sin (lunar day)				1.46	0.26 – 7.80	0.647
Observations	40			40		
Pseudo-R^2 Nagelkerke	0.44			0.56		

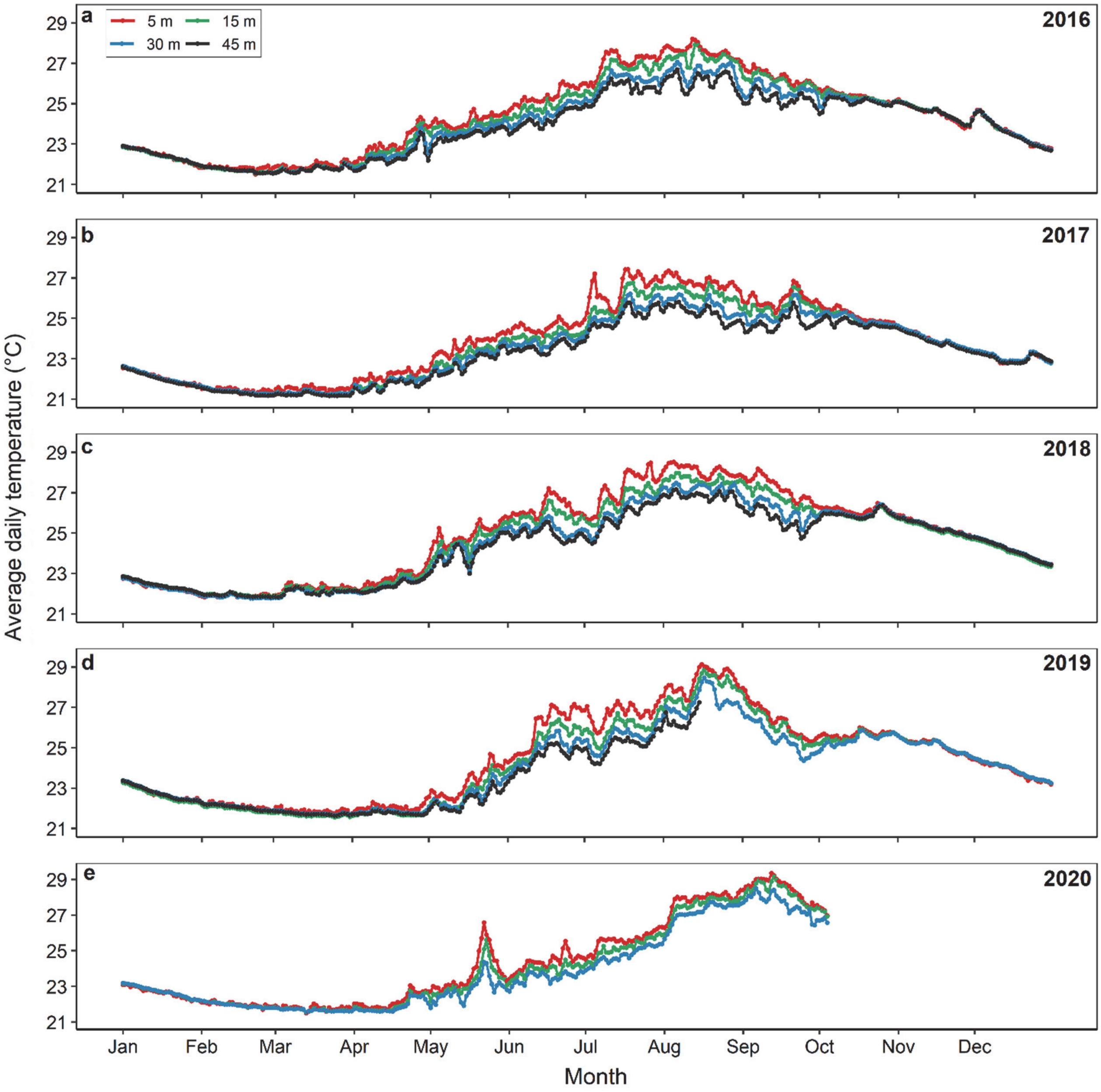

Figure S1. Mean daily seawater temperatures from shallow to mesophotic depths during 2016–2020. The different panels (a–e) represent the different years and the colored lines represent the mean daily seawater temperatures at four different depth zones according to the legend in (a).

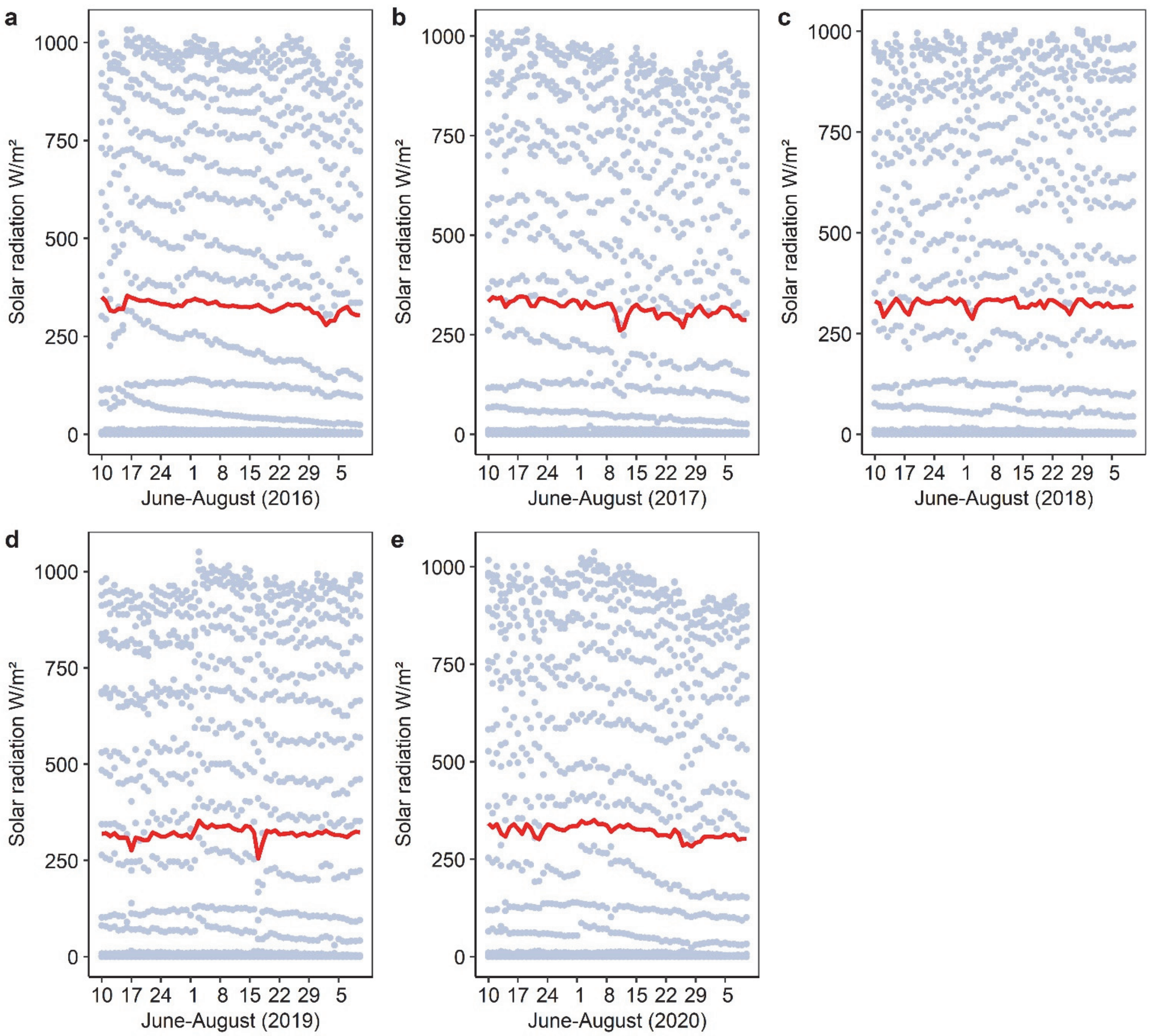

Figure S2. Daily solar irradiance records at the Interuniversity Institute for Marine Sciences Eilat (IUI) during June–August, 2016–2020. The different panels (a–e) represent the different years and the red lines represent the mean daily solar radiation.

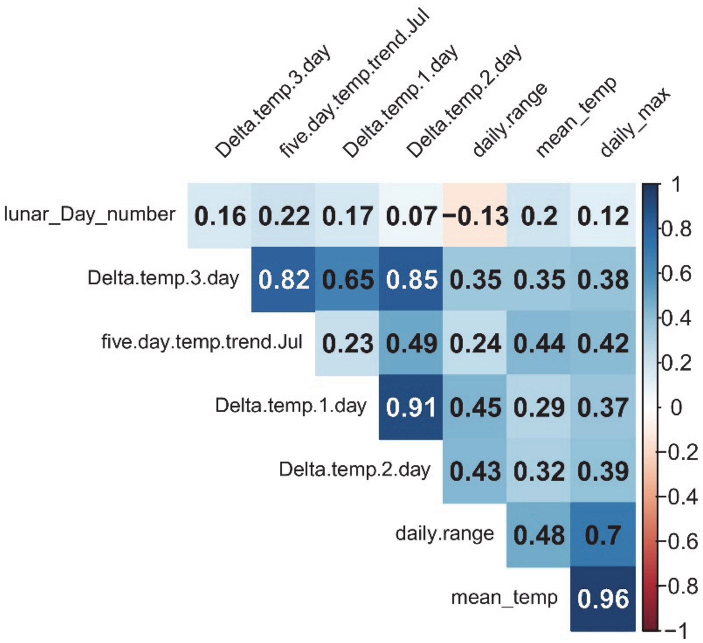

Figure S3. Pearson correlation coefficients (r) of the relationship between the continuous predictors used in the data analysis procedure.
